# The Role of ApoE in HCV Infection and Comorbidity

**DOI:** 10.3390/ijms20082037

**Published:** 2019-04-25

**Authors:** Yue Gong, Wei Cun

**Affiliations:** Institute of Medical Biology, Chinese Academy of Medical Sciences & Peking Union Medical College, 935 Jiaoling Road, Kunming 650118, China; gongyue.1@foxmail.com

**Keywords:** apolipoprotein E (ApoE), hepatitis C virus (HCV), lipo-viro-particles (LVPs), chronic infection, immune evasion, HCV-associated comorbidities

## Abstract

Hepatitis C virus (HCV) is an RNA virus that can efficiently establish chronic infection in humans. The overlap between the HCV replication cycle and lipid metabolism is considered to be one of the primary means by which HCV efficiently develops chronic infections. In the blood, HCV is complex with lipoproteins to form heterogeneous lipo-viro-particles (LVPs). Furthermore, apolipoprotein E (ApoE), which binds to receptors during lipoprotein transport and regulates lipid metabolism, is localized on the surface of LVPs. ApoE not only participate in the attachment and entry of HCV on the cell surface but also the assembly and release of HCV viral particles from cells. Moreover, in the blood, ApoE can also alter the infectivity of HCV and be used by HCV to escape recognition by the host immune system. In addition, because ApoE can also affect the antioxidant and immunomodulatory/anti-inflammatory properties of the host organism, the long-term binding and utilization of host ApoE during chronic HCV infection not only leads to liver lipid metabolic disorders but may also lead to increased morbidity and mortality associated with systemic comorbidities.

## 1. Introduction

Blood is one of the main routes of viral transmission. During the long history of transmission, many viruses have acquired the ability to target organs using blood components as carriers. It is well known that hydrophobic lipids are transported in human blood and must bind to apolipoproteins to form hydrophilic lipoproteins that can be recognized by specific enzymes and receptors [1]. Under normal physiological conditions, apolipoprotein E (ApoE) acts as an apolipoprotein that is located on the surface of lipoproteins and regulates lipid transport and metabolism between the liver and peripheral tissues by recognizing cell surface receptors [2,3]. In addition, ApoE can affect the antioxidant and immunomodulatory/anti-inflammatory properties of the organism [4]. A large number of experimental results suggest that ApoE is an essential element in the production of infectious Hepatitis C virus (HCV) particles. ApoE is hijacked by the virus during HCV infection and plays an important role. Because ApoE is versatile and is polymorphic within the population, in addition to increasing the complexity of viral infection, the long-term hijacking and utilization of ApoE by HCV also leads to more complex pathological results.

## 2. The Morphological Association of ApoE and HCV LVPs

### 2.1. The Function of ApoE Dictated by Its Structure

**Figure 1:**
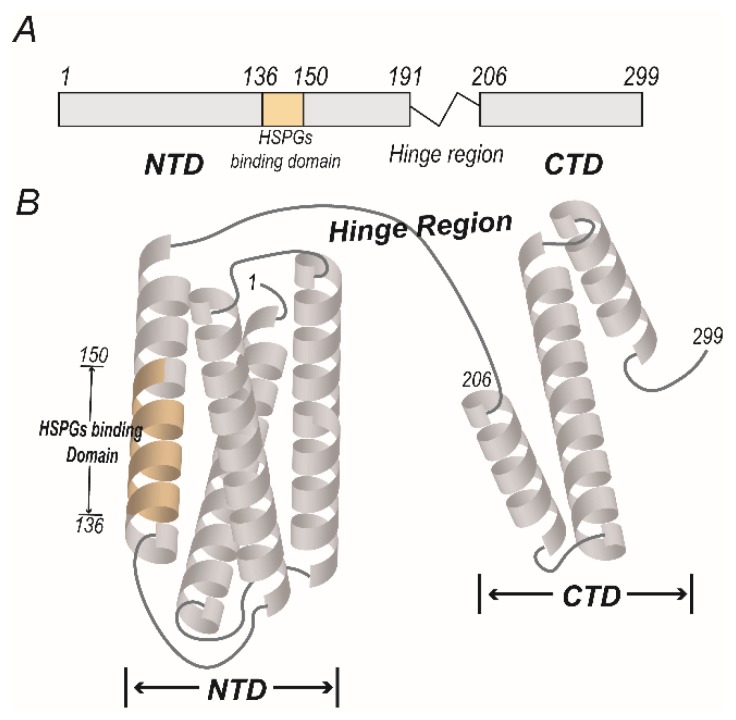
The functional domains of apolipoprotein E (ApoE). (**A**) The protein primary structure of ApoE. The mature ApoE protein contains 299 amino acids, with a hinge region linking two functional domains. The N-terminal domain (NTD) includes residues 1–191, and the cell surface HSPGs binding domain includes residues 136–150. The C-terminal domain (CTD) includes residues 206–299. (**B**) The model of the molecular structure of lipid-free ApoE. The four antiparallel α-helices of NTD form a bundle, and the cell surface HSPGs binding domain lies in the fourth α-helix. The CTD of ApoE consists of folded α-helices and is associated with the lipid binding domain.

ApoE is mainly expressed in the brain, endocrine tissues and liver, and circulated in the blood. In the blood, the ApoE is primarily synthesized by the liver and is a constituent of chylomicrons, chylomicron remnants and lipoproteins like very low-density lipoprotein (VLDL), intermediate-density lipoprotein (IDL), and a subgroup of high-density lipoprotein (HDL) [5]. The mature ApoE protein contains 299 amino acids after the signal peptide is cleaved, and a hinge region links the N- and C-terminal regions (Figure 1A). The N-terminal domain (aa 1–191) consists of a bundle composed of four antiparallel α-helices that contains a binding domain for cell surface receptors (Figure 1B), such as low-density lipoprotein receptors (LDLRs), very low-density lipoprotein receptors (VLDLRs), ApoE receptor 2 (ApoER2) and lipoprotein receptor-related protein 1 (LRP1) [6], with the basic amino acid residues in aa 136–150 of the N-terminal region forming a positively charged fragment that can bind to cell surface heparan sulfate proteoglycan (HSPG) [7]. The principle lipid-binding region lies in the C-terminal domain (aa 206–299) and mainly consists of amphipathic α-helices. ApoE can switch between lipid-associated and lipid-free states [8]. In the lipid-free state, the two functional domains are independently folded and the α-helices of C-terminal regions form a large exposed hydrophobic surface and interact with residues in the N-terminal helix bundle domain through hydrogen bonds and salt bridges. Besides, the affinity of ApoE to LDLRs is low in the lipid-free state but becomes high when associated with lipid, and the changes of affinity may be due to the large conformational changes of ApoE in ApoE-lipid complex and the size, lipid composition and the presence of other apolipoproteins of the complex [8]. Thus, ApoE is an exchangeable apolipoprotein that can be either free or bound to lipoproteins.

### 2.2. HCV Infection

**Figure 2:**
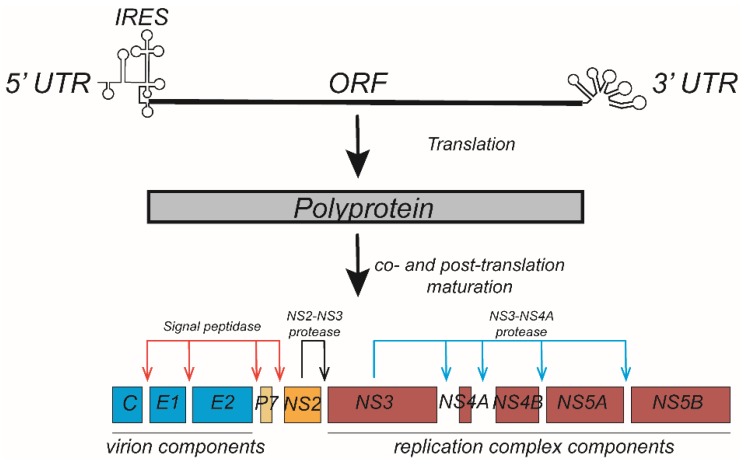
Hepatitis C virus (HCV) genome translation and polyprotein processing. The HCV genome contains an open reading frame flanked by 5′ and 3′ untranslated regions (UTRs) and encodes an approximately 3000 aa polyprotein. Translation of the polyprotein is mediated by an internal ribosome entry site (IRES) within the 5′UTR and is processed into ten viral proteins through a co- and post-translation maturation process. The virion components (core, E1, and E2 proteins) and p7 are cleaved from the polyprotein by cellular signal peptidase. NS2 is cleaved from NS3 by the protease activity of NS2, while the viral replication components (NS3-NS5B) are cleaved from one another by the NS3-4A protease.

HCV is a hepatotropic enveloped single-stranded RNA virus of the Flaviviridae family for which the highest transmission efficiency occurs through blood. The HCV genome contains 5′ and 3′ noncoding regions and a single ORF that is translated into a polyprotein. The polyprotein is co- and post-translationally processed by cellular (signal peptidase) and viral proteases (NS2 autoprotease and NS3 protease) to generate ten viral proteins (Figure 2). The viral structural proteins, including the nucleocapsid protein (core) and two envelope proteins (E1 and E2), are involved in the assembly of the HCV particle. The most of nonstructural proteins (NS3, NS4A, NS4B, NS5A, and NS5B) that are involved in viral genome replication [9]. In addition, p7 and several nonstructural proteins (NS2, NS3, NS4A and NS5A) are also involved in the assembly of the viral particle. During infection, HCV particles are trapped by HSPGs and internalized into the cytoplasm through endocytosis upon interaction with various receptors, including SR-BI, CD81, claudin-1, and occludin [10]. Acute infections occur immediately after individuals become infected by HCV, but it is difficult for acutely infected patients to achieve self-cure, and more than half of these patients will develop chronic infections that are associated with a greater chance of liver fibrosis, cirrhosis, and hepatocellular carcinoma [11]. The mechanism of how HCV induces chronic infection is still unclear, in addition to the rapid mutation of the HCV genome, the close association of the HCV replication cycle with host lipid metabolism likely contributes to chronicity of infections.

### 2.3. ApoE Is a Morphological Component of the HCV LVPs

**Figure 3:**
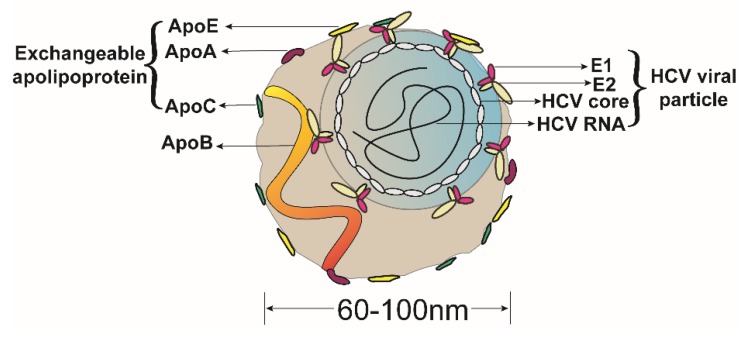
Structure of the HCV LVPs. The HCV LVPs consists of an HCV viral particle (blue circle) and lipid components (brown shape). The heterodimer of HCV envelope proteins 1 and 2 (E1 and E2) are embedded in the endoplasmic reticulum membrane to form the HCV envelope, after which they encapsulate the HCV nucleocapsid, which consists of the HCV core protein and HCV RNA, ultimately generating the HCV particle. The lipid components are rich in triglycerides and cholesterol and are associated with lipoprotein-associated protein components, such as apolipoproteins, including ApoA, ApoB, ApoC and ApoE. The ApoE on the surface of HCV LVPs may mask the viral E1E2. The HCV particle and lipid components interact in the endoplasmic reticulum during the assembly process. The HCV LVP is irregular in shape and heterogeneous in density, with a diameter between 60 and 100 nanometers.

Shortly after the discovery of HCV, researchers observed that the HCV genome was distributed over a wide range of densities by density gradient centrifugation of patient sera, with the lower density components being highly infectious [12,13], which is probably due to the binding of HCV virions to lipoproteins in patient blood to form LVPs. Because the density of these LVPs is heterogeneous [14], after being analyzed by immunoprecipitation and proteomics, it was speculated that the LVPs may contain various apolipoproteins, such as ApoA, ApoB, ApoC, and ApoE [15]. With the establishment of a robust HCVcc (HCV cell culture) system [16,17,18,19], Gastaminza et al. used immunoelectron microscopy to first observe the presence of ApoE on the surface of concentrated HCVcc particles that were produced from Huh-7.5.1 cell [20]. Merz et al. used affinity chromatography to purify HCV particles with an E2^flag^, and ApoE was also observed on the surface of HCV LVPs [21]. Catanese et al. purified HCVcc particles with affinity grids and observed the presence of some ApoB and multiple copies of ApoE and ApoA1 on the rough surfaces of intact HCV LVPs [22]. Interestingly, on the surface of HCV particles, host apolipoproteins were more readily accessible to antibody labeling than HCV glycoproteins, suggesting that the latter were either present at a lower abundance or were masked by host proteins. Recently, based on the direct specific immunocapture of particles on transmission electron microscopy grids, Piver et al. observed that the nucleocapsid of HCV is surrounded by an irregular, detergent-sensitive lipid. HCV circulates in the serum of patients as part of a mixed population of putative LVPs and lipoprotein-like particles, and at least a fraction of viral particles (putative LVPs) display ApoB, and ApoE on their surface [23]. The presence of apolipoproteins on the surface of HCV virions (Figure 3) suggests that apolipoproteins, such as ApoE, may be involved in the assembly and infectivity modification of HCV infectious virus particles.

## 3. Role of ApoE in the HCV Infection

### 3.1. Apolipoprotein E Is Required for Production of HCV

The presence of several apolipoproteins on the surface of HCV LVPs suggests that apolipoproteins may play a role in HCV production. The assembly of infectious HCV virions requires efficient viral genome replication, and when the HCV genome is released from the HCV replication complex, in addition to viral proteins, the cellular host factors are also involved in viral particle assembly. The HCVcc infectious particle is produced by the most-used JFH-1 (genotype 2a) HCVcc system [16,17,18,19], thus most of our understanding of the role of ApoE in HCV production are based on HCV genotype 2a strain and its derivatives. Moreover, the expression of ApoE did not affect the replication efficiency of HCV genotype 2a in the HCV replicon system, though the ApoE may affect the replication of the HCV genome of the genotype 1b [24]. In the HCVcc system, downregulating the expression of ApoE by small interfering RNA (siRNA) interference significantly reduced the production of infectious HCV, however, knockdown of other apolipoproteins did not significantly inhibit HCV production, suggesting that ApoE plays an essential role in HCV production [25]. Further, the knockdown of ApoE decreased the production of extracellular HCV particles but did not affect the formation of intracellular viral nucleocapsids or the expression of the envelope proteins, suggesting that ApoE is involved in a late step in HCV assembly [26,27]. The inhibition of ApoE expression can significantly reduce the production of infectious HCV particles in a dose-dependent manner, and the exogenous expression of a truncated protein containing the CTD of ApoE can restore HCV production, indicating that the CTD of ApoE is involved in HCV assembly and release [28,29,30]. Further studies have shown that the interface between lipid droplets and the endoplasmic reticulum is the HCV assembly platform; in this process, the binding of the NS5A protein and the autophagy system facilitate the recruitment of ApoE to the virion assembly site [31,32], while the CTD of ApoE participates in HCV production by binding to the HCV envelope proteins [26,33,34]. Due to the lack of efficient cell culture systems for other major HCV genotypes, the investigation of genotype-specific influences of ApoE on the production of the viral infectious particle is based on the JFH1-chimeras. The results from Huh7.5 and 293T cells showed that the ApoE affects the production of the JFH1-derived hybrid virus with different genotype structural gene in a strain-dependent manner and is more efficient than other exchangeable apolipoproteins in the assembly of viral particles in all tested strain [35].

In apolipoprotein-deficient cells, such as mouse immortalized hepatocytes, Vero cells and 293T cells, the ectopic expression of ApoE is essential for the efficient production of infectious HCV virions [36,37,38]. In contrast, the knockout of ApoE in Huh7 hepatoma-derived cells expressing several apolipoproteins by using a gene editing system did not completely inhibit HCV production [35,39,40] [34,35,40]. Thus, the CTD of ApoE is a significant factor involved in the assembly of HCV infectious particles, but it is not the only factor. First, the species specificity of ApoE is not crucial for the formation of infectious HCV virions. Murayama et al. demonstrated that a monkey kidney cell line ectopically expressing human ApoE was capable of supporting the production of HCVcc [37]. Moreover, another group showed that ApoE in both mice and humans can support the HCV assembly with comparable efficiency in mouse hepatoma cell lines [38]. Second, those proteins containing amphipathic α-helices, including other apolipoproteins, such as ApoC1, human cathelicidin antimicrobial peptide, and two viral secretory glycoproteins E^rns^ and NS1 from the Flaviviridae family members pestivirus and flavivirus respectively, can also be substituted for ApoE to participate in the assembly of HCV particles [40,41,42]. Fukuhara et al. demonstrated the role of amphipathic α-helices of exchangeable apolipoproteins in the formation of HCV infectious particles [40]. Furthermore, the exogenous expression of E^rns^ and NS1 could compensate for the absence of apolipoproteins in HCV particle formation in ApoB/E double-knockout Huh7 cells and 293T cells, indicating that the viral glycoprotein has an overlapping function with ApoE [41]. Therefore, HCV can use a variety of host apolipoproteins and viral glycoproteins as a substitute for ApoE.

### 3.2. HCV Infectivity Is Influenced by ApoE

**Figure 4:**
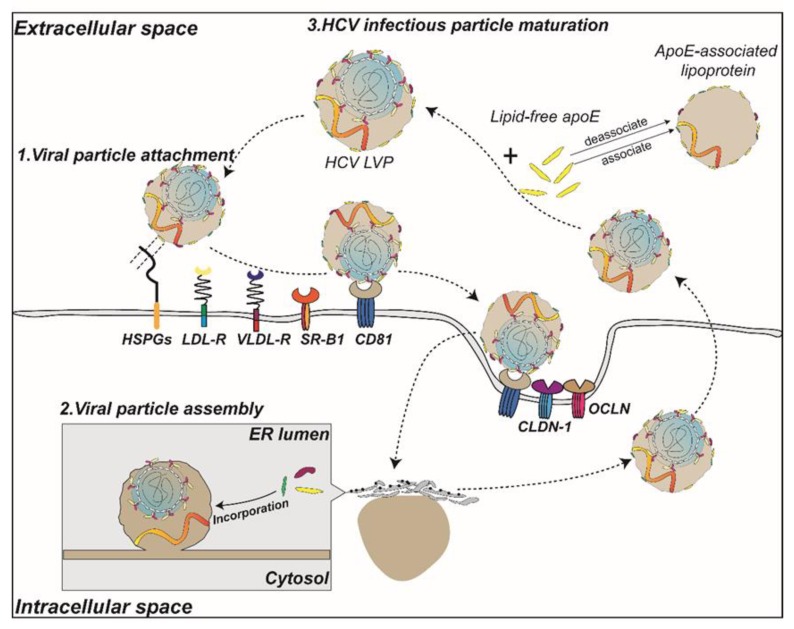
A model of the role of ApoE in the life cycle of HCV. The circulating HCV infectious particles bind to HSPGs and receptors (LDLr, VLDLr, and SR-B1) on the cell surface via ApoE prior to entering cells via interactions with CD81, claudin-1 (CLDN-1) and occludin (OCLN). Subsequently, in the lumen of the endoplasmic reticulum, apolipoproteins such as ApoE are incorporated into lipid droplets that encapsulate the HCV nucleocapsid, which is embedded with E1E2 heterodimers to participate in the assembly process. In addition, the binding of extracellular ApoE to secreted HCV particles assists in the maturation of HCV LVPs.

The N-terminal domain (NTD) of ApoE mediates lipoprotein recognition by multiple receptors (Figure 4). The use of ApoE antibodies or competitive peptides of ApoE receptor binding regions can efficiently block HCV infection of hepatocytes, suggesting that the NTD of ApoE is involved in HCV binding or entry into hepatocytes [43,44,45]. Increasing the positive charge of the heparin-binding region (aa 136–150) of the ApoE NTD can enhance the infectivity of HCV, whereas treating HSPGs with heparinase can block the attachment of HCV to hepatocytes, suggesting that HCV may utilize the charge adsorption capacity between ApoE and HSPGs to mediate HCV attachment to hepatocytes [39,43,46]. Among these HSPGs, syndecan-1 or syndecan-4 have been reported to be the heparan sulfate proteoglycans that mediate the attachment of HCV [47,48]. It has also been reported that HCV can enter hepatocytes by the binding of ApoE and scavenger receptor B1 (SR-BI), a receptor on the surface of hepatocytes [49].

Whether the interaction between ApoE and other cell surface receptors can also mediate HCV entry into cells remains controversial. However, ApoE is known to recognize LDLr or VLDLr on the surface of hepatocytes and mediate cholesterol and lipid transport into hepatocytes [8,50]. Several studies have shown that HCV LVPs attach to hepatocytes through the binding of ApoE and LDLr [51], although other studies have shown the opposite results [52]. Because the oxygen content of in vitro cell culture systems is known to differ from the liver in vivo [53], studies based on normoxic conditions in vitro cell models may ignore the role of VLDLr. Recently, some studies have shown that the expression of VLDLr under hypoxic conditions or the exogenous expression of VLDLr is involved in mediating HCV entry into hepatocytes, and this interaction requires both HCV E2 and ApoE [54,55].

ApoE gene polymorphisms have been observed among the HCV population [3]. There are three major isoforms (E2, E3, and E4) that differ by the relative charge, and many rare isoforms are also known to exist [56]. In the different genotype HCV pseudoparticles (HCVpp) which are formed by incorporation of the full-length E1 and E2 proteins onto lenti-or retroviral nucleocapsids [57], the infectivity of HCVpp is affected by the different ApoE isoforms [39]. Moreover, the different affinities of the various ApoE isoforms for cell surface receptors can affect host susceptibility to HCV infection and the ability to clear the virus after infection [58]. A 2006 survey showed that individuals with the ApoE-ε3 allele are associated with persistent infection, whereas the ApoE-ε2 allele are associated with the equivalent of a 3–5-fold reduction in the risk of chronic HCV infection [59]. Another study also demonstrated that the exogenous expression of ApoE2 protein poorly compensates for the production of HCV-infected particles in ApoE knockout cells, whereas the exogenous expression of ApoE3 and ApoE4 can restore this production to almost normal levels [29]. Several recent surveys have also suggested that individuals carrying the ApoE-ε2 allele are resistant to HCV infection, while the ApoE-ε4 allele is beneficial for viral clearance after HCV infection and recovery after combination therapy. However, the ApoE-ε3 allele is considered to be a particular risk factor for the establishment of chronic infection after HCV infection [60,61]. The effect of different ApoE isoforms on HCV infectivity suggests that the binding of ApoE to HCV can modulate viral infectivity, resulting in different HCV infection efficiencies in individuals carrying different ApoE alleles.

### 3.3. ApoE Is Essential for Efficient Cell-to-Cell Transmission of HCV

In addition to cell-free particle diffusion, HCV can also spread through a direct cell-to-cell transmission route that is dependent on Niemann-Pick C1-Like 1, claudin-1, and occludin [9]. Hueging et al. observed in 293T/mir122 cells that the spread of HCV toward recipient cells only occurs in a coculture of donor cells expressing ApoE, demonstrating that donor cells lacking ApoE could not mediate intercellular transmission of HCV, suggesting that ApoE is involved in the process of HCV transmission between cells [27]. Gondar et al. observed the cell-to-cell spread was compromised in donor, but not in recipient cells, when ApoE was knocked down rather than ApoB, indicating that ApoE in donor cells is required for HCV intercellular transmission in cells [62]. In addition, the results of another study showed that knockdown of ApoE by siRNA in Huh7 cells did not prevent HCV intracellular transmission but did reduce the size of foci [63].

### 3.4. Alteration of HCV Infectivity by Extracellular ApoE

To adapt to the extracellular environment or to enhance their infectivity, some enveloped viruses undergo particle modification during their release to the extracellular environment. ApoE is an exchangeable apolipoprotein that exists in either a lipid-free or lipoprotein-associated state in the blood, and in the lipoprotein-associated state can load lipid to regulate the lipid metabolism [8]. HCV is known to be efficiently transmitted through the blood. The structure of HCV LVPs is not uniform, and the ApoE copy number on the surface of HCV LVPs is variable [23]. To adapt to the host lipid metabolism or when infecting with new hosts, HCV LVPs also interact with ApoE in the blood environment when released from hepatocytes into the blood (Figure 4).

In recent years, several studies have shown that during HCV infectious particle production, HCV intracellularly binds ApoE and can also bind or exchange ApoE in the cell culture supernatant or blood with the viral envelope protein when released into the extracellular environment [39,64,65,66]. This ability can further increase the abundance of ApoE on HCV LVPs, thereby enhancing the infectivity of HCV. On the other hand, HCV can further adapt to the host lipid environment in the blood and the different ApoE isoforms to achieve immune evasion.

Under in vitro culture conditions, the infectivity of HCV can be altered with secreted ApoE (sApoE) of different isoforms in the cell culture medium. When using equal amounts of HCVcc particles, the infectivity of HCV LVPs can be enhanced by using a low concentration of different isoforms of sApoE, although a high concentration of sApoE can competitively inhibit HCV infectivity, indicating that the ability of HCV LVPs to bind ApoE can be saturated [39]. The concentration of ApoE secreted from Huh7 cells to the supernatant is only 0.1–0.5 mg/mL, whereas the normal physiological concentration of ApoE in the serum is 10–60 mg/mL. Since different concentrations and isoforms of ApoE among individuals do exist, the infectivity of HCV in vivo could be affected by the ApoE in the blood.

The replication of HCV in the liver and its interaction with intracellular ApoE only affects the liver lipid metabolism, but the long-term release of HCV LVPs into the blood and its persistent interaction with extracellular ApoE suggests that HCV may directly trigger systemic lipid metabolic comorbidities.

## 4. Function of ApoE and HCV-Associated Comorbidities

### 4.1. ApoE Promotes Immune Evasion for HCV Chronic Infection

HCV antibodies are one of the main indicators for the clinical detection of HCV infection. High levels of neutralizing antibodies can be detected in the blood of patients with chronic hepatitis C, but they are not able to control the viremia, nor can they prevent HCV from spreading to the new host through blood. The high frequency of mutation of the viral genome is a mechanism by which viruses escape the immune system [67,68], but the mutation of RNA viruses alone is not sufficient to establish chronic infections. In the presence of neutralizing antibodies (NAbs), low-density fractions of serum-derived HCV can also be transmitted, suggesting that the lipoprotein coats of HCV are the predominant means by which the virus evades the host immune system [13]. 

Sheridan et al. observed that the complete early virological response (EVR) toward HCV genotype 1 was associated with lower ApoE compared to null responders, suggesting that high concentrations of ApoE can reduce host responsiveness to interferon [69]. Ficolin-2 (L-ficolin/p35) is a lectin-complement pathway activator that neutralizes and inhibits the initial attachment and infection of HCV by binding to the HCV surface envelope glycoproteins E1 and E2, regardless of the viral genotype. The interaction between ApoE3 and viral particles blocks the effect of ficolin-2 and mediates an immune escape mechanism during chronic HCV infection [70].

ApoE is utilized in the HCV cell-to-cell transmission mechanism, which is another route that mediates HCV infectious particle escape from the NAbs response [71]. A recent study demonstrated that the incorporation of extracellular secreted ApoE into HCV LVPs enhanced the interaction of the particles with cellular HSPGs, subsequently reducing the neutralizing effect of NAbs toward HCV [65]. The mutation of the HCV E2 protein led to a decrease in binding to low-density lipoprotein or VLDL and increased the sensitivity of HCV to NAbs [72]. Moreover, another study also observed that the conformational epitope domains (B and C) that lie in the HCV E2 protein were exposed after depletion of ApoE [73]. This result suggests that the neutralizing epitopes of HCV are shielded by the ApoE localized on the surface of HCV LVPs. Therefore, ApoE contributes to the ability of HCV to evade the effects of innate immunity and adaptive immunity, which makes it difficult for HCV to be cleared and promotes the establishment of a chronic infection that leads to persistent liver inflammation.

### 4.2. Hints Regarding Persistently Hijacked ApoE and HCV-Associated Comorbidities

In addition to hepatitis, HCV chronic infection can trigger glycolipid-associated metabolic syndrome. Clinical data show that HCV carriers are more likely to develop type 2 diabetes than healthy individuals, and the age of onset is significantly advanced [74,75]. In HCV chronic infection patients, the carotid intimal media thickening is increased 4.03-fold compared with uninfected individuals, and the probability of forming carotid plaque is increased by 3.94-fold [76]. Furthermore, the incidence of atherosclerosis was significantly increased compared with the control group [76,77,78]. Clinical evidence indicates that chronic HCV infection is associated with a dysregulation of circulating lipoproteins and apolipoproteins [79,80,81]. Since the liver is a vital metabolic organ, these metabolic syndromes may be comorbidities of chronic hepatitis caused by HCV. However, the association between chronic hepatitis induced by Hepatitis B Virus infection and glycolipid-associated metabolic syndrome is not significant [82], suggesting that chronic hepatitis may not be the primary cause of metabolic imbalances.

**Figure 5:**
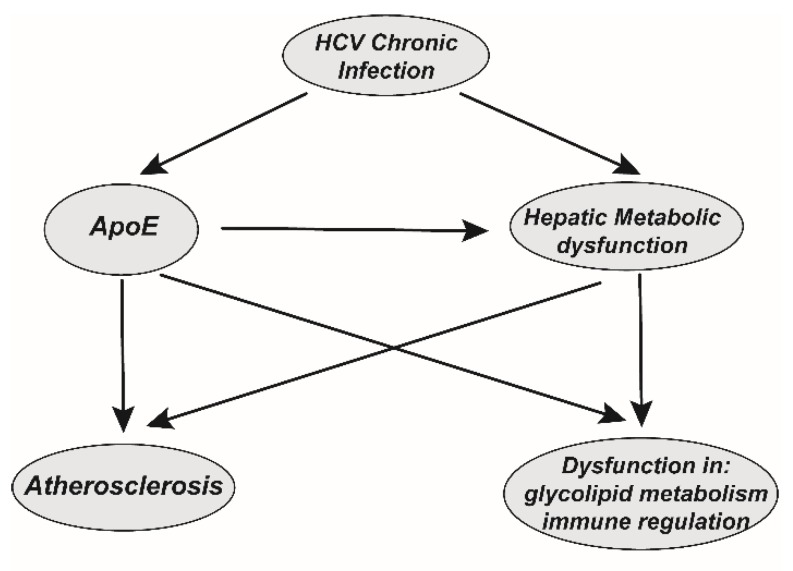
The potential correlation between HCV chronic infectious and HCV-associated comorbidities. HCV chronic infection induces persistent hepatitis and liver metabolic dysfunction. In addition, HCV participates in the long-term hijacking of ApoE, which further promotes the dysfunction of liver metabolic function. On the one hand, liver metabolic dysfunction caused by chronic infection may lead to systemic dysfunction of glycolipid metabolism. On the other hand, ApoE deficiency is associated with atherosclerosis and glycolipid metabolism and immune regulation dysfunction. Thus, persistent hijacking of ApoE by chronic HCV infection may potentially contribute to these diseases and may be the potential cause of comorbidities associated with chronic HCV infection.

The importance of ApoE in lipid metabolism was recognized decades ago. In animal models, ApoE deficiency is associated with atherosclerosis [81], while ApoE deficiency can also result in a reduction in anti-inflammatory M2 macrophages [4]. Therefore, persistent sApoE is hijacked by HCV in the extracellular environment, which may lead to abnormalities, such as insulin resistance in the pancreas and the formation of foam cells in the vascular intima, which may further promote metabolic tissue dysfunction. The assembly, release, infection and immune evasion of HCV can be modulated inside or outside of hepatocytes by the binding or utilization of the host ApoE. Thus, in the long-term chronic infection process HCV causes the abnormal function of ApoE, and investigation of the relationship of these factors may help us understand the cause of HCV-associated metabolic syndrome (Figure 5). However, whether this process leads to HCV-associated metabolic syndrome requires further research.
